# Potential of Vagus Nerve Stimulation to Modulate Fibromyalgia’s Network Physiology: A Systematic Review

**DOI:** 10.3390/jfmk11010015

**Published:** 2025-12-29

**Authors:** Joao Pedro Perin, Carla Pastora-Sesín, Sungjoon Kang, Alba Navarro-Flores, Felipe Fregni, Kevin Pacheco-Barrios

**Affiliations:** 1 Neuromodulation Center and Center for Clinical Research Learning, Harvard Medical School, Spaulding Rehabilitation Hospital, Boston, MA 02138, USA; 2 Trybe Health, Torre Lexus, Avenida Escazú, San Rafael de Escazú 10203, Costa Rica; 3 Admission AG, Irvine, CA 92618, USA; 4 Institute of Psychiatric Phenomics and Genomics (IPPG), LMU University Hospital, LMU Munich, 80336 Munich, Germany; 5 International Max Planck Research School for Translational Psychiatry (IMPRS-TP), 80804 Munich, Germany; 6 Unidad de Investigación para la Generación y Síntesis de Evidencias en Salud, Vicerrectorado de Investigación, Universidad San Ignacio de Loyola, Lima 15023, Peru

**Keywords:** fibromyalgia, vagus nerve stimulation, autonomic regulation, neuroinflammation, network neuroscience

## Abstract

**Background**: Chronic pain conditions such as fibromyalgia syndrome (FMS) reflect maladaptive network physiology across perceptual–autonomic–immune axes, yet most treatments remain symptomatic and incompletely effective. **Methods**: We conducted a comprehensive systematic review to evaluate vagus nerve stimulation (VNS) and FMS within a network physiology framework. PubMed, Embase, and Cochrane CENTRAL were searched on October 24, 2025. Risk of bias was assessed using the ROB-2 tool. An iterative thematic synthesis was performed to develop an integrative conceptual framework and to identify knowledge gaps and future research directions. **Results**: We first summarize physiological evidence showing autonomic imbalance (e.g., decreased heart rate variability), neuroinflammatory activation, and aberrant cortical network connectivity in FMS, supporting a network-dysregulation model. We then included 6 studies (4 clinical studies and 2 protocols) on VNS effects, highlighting improvements in pain, fatigue, sleep disturbance and autonomic regulation, along with emerging mechanistic insights. Key methodological heterogeneity—such as stimulation parameters, outcome metrics, type of control arm, sham definition, and small samples—limits current interpretability. Finally, we outline a research agenda centered on network-based biomarkers, immunophenotyping, adaptive trial designs and stratification of responders, with the aim of validating taVNS as a scalable neuromodulatory intervention for FMS. **Conclusions**: By reframing FMS from a symptom-centric pharmacologic model to a network-centric neuromodulation approach, taVNS is a promising tool for mechanism-based therapeutics in central sensitization syndromes and chronic pain.

## 1. Introduction

Fibromyalgia syndrome (FMS) is a chronic, complex disorder characterized by widespread musculoskeletal pain, fatigue, non-restorative sleep, cognitive impairment (“fibro fog”), and mood disturbances. FMS exhibits a distinctive epidemiological profile characterized by marked gender disparity and age-dependent prevalence. Globally, FM affects approximately 2–4% of the general population, with consistently higher prevalence estimates reported among women compared with men [1,2]. Female predominance is a defining feature of the condition, with female-to-male ratios ranging from approximately 2:1 to as high as 7–9:1, depending on diagnostic criteria, geographic region, and study methodology [2,3]. The highest incidence occurs in adult women between 20 and 55 years of age, although FMS can affect individuals across the lifespan, including children, adolescents, and older adults [1,2].

Epidemiological data further suggest that prevalence increases with advancing age, with up to 8% of individuals aged ≥80 years meeting diagnostic criteria in some population-based studies [2]. While women on average report greater symptom severity and functional impairment, emerging evidence indicates that FMS in men may be underdiagnosed due to gender-related diagnostic bias and differences in symptom reporting, potentially leading to underestimation of true male prevalence [3]. Moreover, the presentation of this condition frequently exhibits significant overlap with other central sensitivity syndromes, such as irritable bowel syndrome and chronic fatigue syndrome [4,5], further increasing the complexity of this condition.

The repercussions of FMS extend beyond the manifestation of symptoms. Recent cost-of-illness studies demonstrate that FMS continues to impose a substantial and context-specific economic burden across healthcare systems, largely driven by productivity losses and sustained healthcare utilization. A systematic review synthesizing international evidence estimated annual per-patient direct healthcare costs ranging from approximately €1200 to €8500 in European countries, with substantially higher total societal costs once indirect costs, particularly absenteeism and reduced work capacity, were considered [6]. More recent population-based analyses further underscore this burden. In Denmark, registry-based data indicate a net excess societal cost of approximately €27,000 per patient-year following diagnosis, primarily attributable to loss of income, disability pensions, and increased public transfers [7]. At the national level, a 2024 Spanish study estimated the annual social costs attributable to FMS to range between €2.4 and €7.2 billion (2021 values), with informal care and labor productivity losses accounting for the majority of expenditures [8]. Collectively, these contemporary data highlight that the economic impact of FMS remains profound, country-dependent, and predominantly driven by indirect costs related to work disability and caregiving demands.

Consequently, the therapeutic options available to address this condition remain limited. Current clinical guidelines, including those from the European League Against Rheumatism (EULAR) and the American College of Rheumatology (ACR), recommend a multimodal approach with emphasis on non-pharmacological strategies such as patient education, aerobic exercise, and cognitive-behavioral therapy as first-line interventions [9]. Pharmacological treatments, including duloxetine, milnacipran, and pregabalin, are typically reserved for cases that are more severe or resistant to other forms of treatment.

However, these medications frequently provide only partial relief and are associated with a range of adverse effects. Recent studies suggest that FMS could be a chronic disease of the nervous system, with reported alterations in small-fiber and brain processes [10,11]. In light of the patient burden, limited pharmacologic efficacy, and rising costs, there is an urgent need for innovative, safe, and sustainable treatment alternatives that target the nervous system. Non-invasive vagus nerve stimulation (nVNS) has recently emerged as a promising bioelectronic intervention for chronic pain conditions. By modulating central pain pathways, reducing neuroinflammation, and restoring autonomic balance, nVNS offers a novel therapeutic strategy that directly addresses some of the underlying mechanisms implicated in FMS.

This paper aims to systematically review the clinical evidence (randomized and non-randomized) of VNS in FMS, explore its effects in the FMS network physiology, assess its risk of bias, and identify gaps and future directions in this emerging field.

## 2. Methods

### 2.1. Search Strategy

We conducted a comprehensive systematic literature search to identify clinical studies evaluating vagus nerve stimulation (VNS), including both invasive and non-invasive modalities, in individuals with FMS. The search was developed in consultation with established systematic review methodology and followed PRISMA recommendations for transparent reporting of evidence identification and selection [12,13]. Searches were performed in PubMed, Embase, and the Cochrane Central Register of Controlled Trials (CENTRAL) from database inception to 24 October 2025. The pre-defined detailed protocol was registered in Open Science Framework and is available at https://osf.io/ea89c (accessed on 22 November 2025).

The search strategy combined controlled vocabulary (MeSH and Emtree terms) and free-text keywords related to FMS and vagus nerve stimulation. Search terms included combinations of *fibromyalgia*, *fibromyalgia syndrome*, *vagus nerve stimulation*, *VNS*, *transcutaneous vagus nerve stimulation*, *auricular vagus nerve stimulation*, *taVNS*, *nVNS*, *cervical vagus nerve stimulation*, and *vagal tone.* The full electronic search strategies were adapted for each database and are available in the Supplementary Materials. No restrictions were applied regarding publication status, and reference lists of included articles and relevant reviews were hand-searched to identify additional eligible studies.

### 2.2. Eligibility Criteria

Studies were selected according to predefined inclusion and exclusion criteria based on the Population–Intervention–Study design framework. Eligible studies included adult participants diagnosed with FMS according to established diagnostic criteria, such as those defined by the American College of Rheumatology. Interventions of interest comprised any form of vagus nerve stimulation, including invasive cervical VNS and non-invasive modalities such as transcutaneous auricular or cervical VNS. We included all clinical study designs, encompassing randomized controlled trials, non-randomized trials, pilot and feasibility studies, as well as published clinical trial protocols. Studies were required to report clinical outcomes relevant to FMS (e.g., pain intensity, fatigue, functional status, sleep quality, or quality of life) and/or physiological or mechanistic outcomes, including measures of autonomic function (e.g., heart rate variability), inflammatory biomarkers, or neurophysiological indices. Only articles published in peer-reviewed journals were considered.

Studies were excluded if they were animal or preclinical investigations, case reports or case series including fewer than five participants, or evaluations of autonomic modulation techniques not involving vagal stimulation (e.g., biofeedback alone). Conference abstracts were also excluded when insufficient methodological detail was available to allow assessment of study design, intervention characteristics, or outcomes.

### 2.3. Study Screening and Selection

All records identified through database searches were imported into a reference management system, and duplicates were removed. Two reviewers independently screened titles and abstracts for eligibility. Full texts of potentially relevant articles were then assessed independently against the inclusion criteria. Discrepancies at any stage were resolved through discussion and consensus, with involvement of a third reviewer when necessary. This two-stage screening process was conducted in accordance with recommended best practices to minimize selection bias.

### 2.4. Data Extraction

Data were extracted independently by two reviewers using a standardized data extraction form. Extracted variables included: study design, sample size, participant characteristics, diagnostic criteria, VNS modality (invasive vs. non-invasive), stimulation site and parameters, treatment duration, comparator conditions (e.g., sham stimulation, usual care), outcome measures, follow-up duration, and main findings. For trial protocols, planned outcomes and methodological features were extracted. Any disagreements in data extraction were resolved by consensus.

### 2.5. Risk of Bias Evaluation

For randomized controlled trials, risk of bias was assessed using the Cochrane Risk of Bias 2 (ROB-2) Excel tool, version 9, which evaluates bias across five domains: randomization process, deviations from intended interventions, missing outcome data, measurement of outcomes, and selection of reported results [14]. Each domain was rated as low risk, some concerns, or high risk of bias, and an overall risk-of-bias judgment was assigned.

### 2.6. Evidence Synthesis

Given the heterogeneity of study designs, stimulation protocols, outcomes, and follow-up durations, a quantitative meta-analysis was not performed. Instead, evidence was synthesized using a narrative thematic approach, as recommended when statistical pooling is inappropriate [15]. Findings were organized thematically across domains relevant to FMS pathophysiology and treatment, including pain and symptom severity, autonomic regulation, inflammatory markers, and mechanistic insights. Particular attention was paid to consistency of effects across studies, methodological limitations, and gaps in the current evidence base. This approach allowed integration of clinical outcomes with emerging mechanistic data, while maintaining methodological rigor and transparency.

## 3. Results

### 3.1. Why Target the Vagus Nerve?

Network physiology provides a systems-level framework for understanding FMS as a disorder of interacting biological networks rather than isolated organ dysfunction. As defined by Ivanov and colleagues, network physiology refers to the dynamic, non-linear interactions among multiple physiological systems (e.g., autonomic, immune, neural), whose coordinated activity enables adaptive function, while their decoupling or rigidity contributes to disease states [16,17]. Within this framework, the vagal anti-inflammatory reflex, originally described by Tracey, represents a specific neuroimmune control circuit whereby afferent vagal fibers detect peripheral inflammatory signals and relay them to the brainstem, triggering efferent vagal output that suppresses pro-inflammatory cytokine release via cholinergic signaling [18,19].

Operationally, these concepts can be quantified using established physiological and neurobiological measures. Recent findings suggest a potential role for the autonomic nervous system (ANS) in the pathophysiology of FMS, with evidence indicating reduced parasympathetic tone and increased sympathetic activity. This autonomic imbalance has been associated with a variety of health concerns, including chronic pain, fatigue, sleep disturbances, cognitive impairment, and mood issues [4]. Patients consistently exhibit decreased heart rate variability (HRV), a reliable surrogate marker of vagal tone, supporting the concept of impaired autonomic flexibility and persistent physiological stress responses in FMS [5].

**Figure 1:**
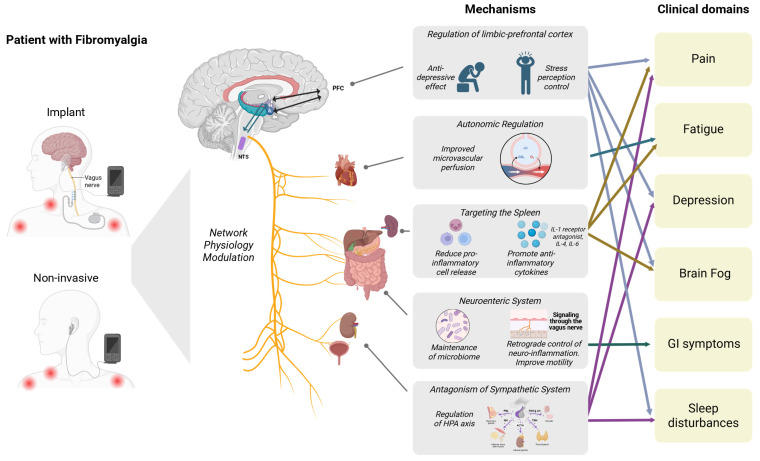
Effects of vagus nerve stimulation on fibromyalgia’s network physiology. Abbreviations: NTS, Nucleus Tractus Solitarius; PFC, Prefrontal Cortex; IL-1, Interleukin-1, IL-4, Interleukin-4, IL-6, Interleukin-6; HPA, Hypothalamic-Pituitary axis; GH, Growth Hormone; TSH, Thyroid-Stimulating Hormone; ACTH, Adrenocorticotropic Hormone; FSH, Follicle-Stimulating Hormone; LH, Luteinizing Hormone; PRL, Prolactin; GI, gastrointestinal.

This autonomic dysregulation aligns with the Polyvagal Theory, which describes the role of the ventral vagal complex in emotional regulation, safety signaling, and social engagement [20]. Chronic suppression of this system can maintain a “defensive” physiological state, amplifying pain perception, anxiety, and emotional dysregulation [20], which is well tested in patients with depression. Beyond these autonomic functions, the vagus nerve plays a main immunomodulatory role through a neuroimmune circuit known as the inflammatory reflex (Figure 1).

Emerging evidence also suggests that the gut–brain axis is a vital part of vagal regulation. This bidirectional system connects the central, autonomic, and enteric nervous systems with the intestinal microbiome, coordinating neural, immune and endocrine signals to maintain gastrointestinal and emotional balance [21]. The vagus nerve is a key communication route within this axis, transmitting sensory information from the gut to the brain and modulating intestinal inflammation and permeability in response to central signals (Figure 1). Alterations in gut microbial composition and barrier integrity have been associated with increased systemic inflammation, heightened pain sensitivity, and greater stress reactivity. This suggests a potential connection between gut dysbiosis, vagal dysfunction, and FMS symptoms [22]. Understanding this interconnected circuitry strengthens the case for targeting vagal pathways to restore neuroimmune balance and autonomic stability in FMS.

Moreover, the inflammatory reflex, first described by Pavlov and Tracey (2012) and expanded by Liu et al. (2024), represents a bidirectional communication pathway between the nervous and immune systems [23,24]. Afferent vagal fibers detect peripheral inflammatory signals and relay them to the nucleus tractus solitarius (NTS) in the brainstem, which coordinates efferent vagal activity through the cholinergic anti-inflammatory pathway (CAP) [9]. This efferent arm inhibits the release of pro-inflammatory cytokines (TNF-α, IL-1β, and IL-6) through acetylcholine-mediated activation of α7 nicotinic acetylcholine receptors (α7nAChR) on macrophages, while stimulating IL-10 production. This reflex enables rapid, localized suppression of inflammation, maintaining immune homeostasis during acute and chronic inflammatory states [19,23] (Figure 1).

Recent work has expanded the understanding of this mechanism with the identification of the vagal–adrenal axis, an additional efferent route where VNS triggers adrenal catecholamine release to dampen systemic inflammation via β_2_-adrenergic receptor signaling [25]. Together, these parallel vagal pathways form a multilayered anti-inflammatory network that bridges neural and immune control.

In FMS, evidence points to immune dysregulation and a chronic low-grade inflammatory state. Elevated concentrations of pro-inflammatory cytokines—including IL-6, IL-8, and TNF-α—and reduced levels of anti-inflammatory mediators such as IL-10 have been reported in both serum and cerebrospinal fluid, correlating with pain amplification, fatigue, and cognitive symptoms [26]. This blunted inflammatory reflex may underlie the persistent activation of pain and stress circuits in FMS.

Critically, these domains are interdependent rather than independent; autonomic imbalance and inflammatory signaling interact with large-scale brain networks to amplify nociplastic pain and symptom persistence. However, translating network physiology into validated biomarkers remains challenging. HRV is sensitive to behavioral and contextual influences, cytokine profiles exhibit substantial inter-individual variability, and neuroimaging markers often lack reproducibility across analytic pipelines. Consequently, while network physiology and the inflammatory reflex offer a coherent mechanistic framework for FMS and vagus nerve stimulation, their current clinical value lies in multimodal integration rather than reliance on any single biomarker—highlighting the need for harmonized protocols and longitudinal systems-level validation.

By re-engaging and modulating this network physiology landscape, VNS emerges as a promising therapeutic approach (Figure 1) that could induce FMS regression or, in some cases, potential remission. nVNS modalities, such as transcutaneous auricular or cervical stimulation, have been shown to reduce cytokine levels, enhance HRV, and modulate pain-related brain connectivity [27,28], suggesting restoration of both autonomic balance and neuroimmune homeostasis. These findings collectively support VNS as a novel therapy capable of addressing the complexity of FMS physiopathology, especially given its favorable safety profile relative to long-term pharmacotherapy.

### 3.2. Clinical Evidence: What Do We Know So Far?

#### 3.2.1. Included Studies

**Figure 2:**
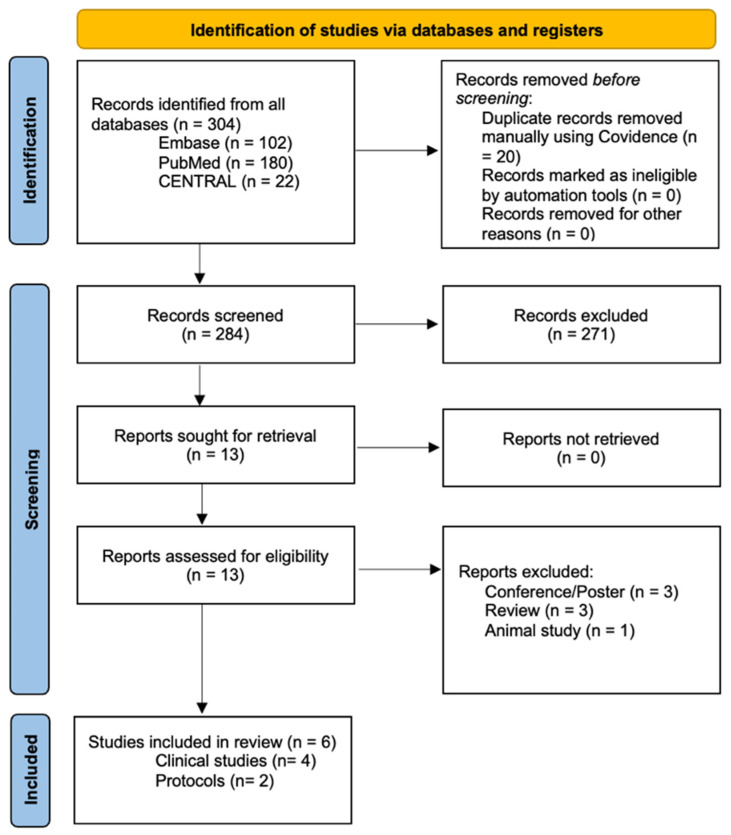
PRISMA flowchart.

The study selection process is summarized in the PRISMA flow diagram (Figure 2). A total of 304 records were identified through database searches, including PubMed (n = 180), Embase (n = 102), and CENTRAL (n = 22). After removal of 20 duplicate records, 284 titles and abstracts were screened, resulting in the exclusion of 271 records that did not meet the inclusion criteria. Thirteen full-text reports were retrieved and assessed for eligibility, all of which were available for review. Of these, six studies met the inclusion criteria and were included in the review, comprising four clinical studies and two published trial protocols.

Table 1 summarizes the key characteristics and findings of the clinical studies evaluating vagus nerve stimulation (VNS) in FMS, including 208 patients. The studies were conducted across the United States, Turkey, Norway, and Italy, with sample sizes ranging from 14 to 116 participants. Study designs were heterogeneous and included an early Phase I/II open-label trial using implanted cervical VNS, pilot and single-arm studies of transcutaneous auricular VNS (taVNS), and a four-arm randomized controlled trial incorporating active and sham taVNS as well as behavioral comparators. Most studies enrolled predominantly female populations, reflecting the epidemiology of FMS. Stimulation protocols varied substantially with respect to modality (most of them auricular non-invasive), stimulation site, frequency, intensity, session duration, and treatment length.

#### 3.2.2. Risk of Bias Assessment

**Figure 3:**
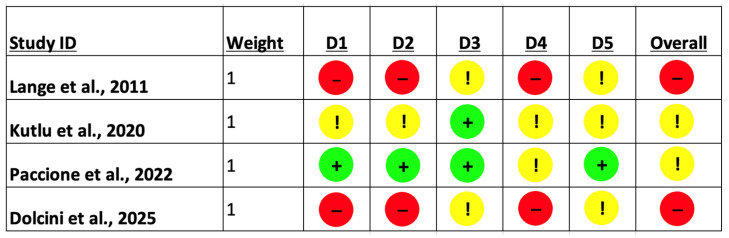
Risk of Bias Assessment [29,30,31,32]. Symbols indicate risk-of-bias judgments for each domain: green circles with a plus sign (+) indicate low risk of bias; yellow circles with an exclamation mark (!) indicate some concerns; red circles with a minus sign (−) indicate high risk of bias.

Risk of bias assessment using the Cochrane ROB 2 tool revealed substantial heterogeneity in methodological quality across the included studies (Figure 3). Early and open-label investigations, such as Lange et al. (2011) and Dolcini et al. (2025), were judged to be at high overall risk of bias, driven primarily by concerns related to the randomization process, deviations from intended interventions, and outcome measurement [29,30]. In contrast, Kutlu et al. (2020) demonstrated some concerns overall, reflecting partial limitations in blinding and outcome assessment despite otherwise acceptable study conduct [31]. Paccione et al. (2022) showed the most robust methodological profile, with low risk of bias across most domains and only minor concerns related to outcome measurement, resulting in an overall judgment of some concerns rather than high risk [32]. Collectively, these findings underscore that while preliminary evidence supports the feasibility of vagus nerve stimulation in FMS, the current evidence base is constrained by methodological limitations, reinforcing the need for larger, rigorously designed, sham-controlled trials with standardized outcome measures.

#### 3.2.3. Individual Studies

Although VNS has been extensively studied in the context of epilepsy and treatment-resistant depression, its application for FMS remains in early stages (Table 1). Nevertheless, a small but growing body of preliminary clinical evidence supports the feasibility and therapeutic promise of VNS—particularly non-invasive modalities—as a novel intervention for FMS [33]. These early investigations, though heterogeneous in methodology and outcome measures, consistently suggest that modulating vagal activity may attenuate core FMS symptoms, including pain, fatigue, sleep disturbances, and mood dysregulation (Table 2).

The first human study to investigate VNS in FMS was conducted by Lange et al. (2011), who performed a Phase I/II open-label trial using implanted left cervical VNS devices in 14 patients with treatment-resistant FM [29]. Despite the invasive nature of the intervention, the study demonstrated encouraging findings: 5 of 12 completers met composite efficacy criteria (improvement in pain, function, and well-being) at 3 months, and some patients no longer met the diagnostic criteria for FM by 11 months of stimulation. Importantly, side effects such as fatigue and dry mouth were reported but were consistent with those observed in other VNS populations (e.g., epilepsy), suggesting acceptable tolerability in FM. This study provided initial evidence that vagal modulation might impact central sensitization and symptom burden in refractory FM cases [29].

Building on this concept with a non-invasive approach, Kutlu et al. (2020) conducted a pilot randomized controlled trial (RCT) comparing transcutaneous auricular VNS (taVNS, 30 min sessions, 5×/week for 4 weeks) combined with home-based exercise versus exercise alone in 60 female patients with FM [31]. Both groups improved significantly in pain, anxiety, depression, and quality of life; however, the aVNS group showed additional improvements in select subdomains of the Short Form-36 (SF-36), including physical function, social functioning, and pain. Although the between-group differences were not statistically significant overall, the authors noted that the aVNS group tended to show larger gains [31]. These findings suggest that auricular VNS may enhance the benefits of exercise through autonomic modulation and central effects on pain perception.

More recently, Dolcini et al. (2025) conducted a single-arm, open-label pilot trial of auricular vagal neuromodulation therapy (AVNT™) in 18 FM patients [30]. Participants received 30 min sessions of taVNS five times per week for four weeks. The intervention led to significant improvements in symptom severity, as measured by the revised Fibromyalgia Impact Questionnaire (rFIQ), as well as enhancements in sleep quality per the Pittsburgh Sleep Quality Index (PSQI). Notably, the study also evaluated serum levels of brain-derived neurotrophic factor (BDNF), hypothesized to reflect neuroplastic or inflammatory changes associated with VNS. However, no significant changes in BDNF levels were observed. This result underscores the complexity of linking clinical improvements with peripheral biomarkers and highlights the need for mechanistic investigations using imaging or neurophysiological tools [30].

A different approach was taken by Paccione et al. (2022), who conducted a four-arm RCT comparing active taVNS, sham taVNS, active meditative-based diaphragmatic breathing (MDB), and sham MDB in 116 adults with severe FM [32]. Participants self-administered treatments for 15 min twice daily over two weeks. While no significant differences were found in the primary outcome—ultra short-term heart rate variability (HRV), a surrogate marker of vagal tone—the authors observed trends toward reduced FM severity in both active treatment groups. Importantly, there was no correlation between HRV changes and pain intensity, suggesting that very short-term HRV measures may be insufficiently sensitive to detect parasympathetic modulation in FM over short intervention periods. Nevertheless, the study reinforces the potential of both behavioral and neuromodulatory interventions to target vagal pathways and improve FM symptoms [32].

In recognition of the need for more robust and methodologically rigorous investigations, the protocol published by Molero-Chamizo et al. (2022) has proposed a large-scale, sham-controlled, parallel-group RCT designed to evaluate the efficacy of non-invasive transcutaneous cervical and auricular VNS in 136 FM patients [34]. The study will include five sessions per week over four weeks and will assess a broad range of clinical and physiological outcomes, including pain intensity, fatigue, sleep quality, depression, heart rate variability, and inflammatory cytokine levels. Notably, the protocol is designed to compare two stimulation sites (auricular vs. cervical), both of which project to the NTS, a central vagal relay involved in pain and affect regulation. This trial represents a critical next step toward clarifying the therapeutic potential of different non-invasive VNS modalities in FM and may help determine optimal stimulation targets and biomarkers of response [34]. A detailed description of the published protocols is presented in Table 3.

Taken together, these early studies and protocols provide foundational clinical evidence that vagus nerve stimulation—whether invasive or non-invasive, auricular or cervical—may beneficially modulate the multidimensional symptom complex of FMS. While findings remain preliminary, they collectively support the hypothesis that impaired parasympathetic regulation plays a role in FM pathophysiology and that restoring vagal tone through neuromodulation may offer a safe, scalable, and mechanism-based treatment strategy.

**Table 1:** Summary of clinical studies on vagus nerve stimulation in FMS.

Study, Year	Country	Study Design	Pain Condition	Sample Size	Mean Age	Percentage of Female (%)	Type of VNS	Stimulation Protocol	Control Group	Main Findings
Lange et al., 2011 [29]	United States	Phase I/II open-label trial	Treatment-resistant FM	14	Range 35–54 (mean not specified)	100%	Implanted cervical VNS.	Continuous stimulation; follow-up to 11 months. Intensity was increased to deliver as high a current as could be comfortably tolerated [target range: 1 to 2 mA]; pulse width = 250 μsec; frequency = 20 Hz; duty cycle = 30 s on, 5 min off.	No control group	5/12 completers improved in pain, function, and well-being; some no longer met FM criteria; side effects (fatigue, dry mouth) tolerable
Kutlu et al., 2020 [31]	Turkey	Pilot RCT (unblinded)	FM	60	39.44 ± 8.28 (active group); 38.60 ± 9.34 (control group)	100%	Transcutaneous auricular VNS (taVNS) via TENS	30 min sessions, 5×/week for 4 weeks (20 total); 10 Hz, biphasic, asymmetrical waveform, pulse < 500 µs, intensity at sensory threshold. Location: bilateral inner/posterior tragus and concha. Weekly check-ins (×4)	Exercise-only control	Both groups improved; taVNS group showed greater (though nonsignificant) improvements in SF-36 physical and social functioning and pain
Paccione et al., 2022 [32]	Norway	Four-arm RCT (planned single-blinded; occurred as double-blinded)	Severe FM	116	45.69 ± 10.25	94.8%	Transcutaneous auricular VNS (taVNS) vs. sham taVNS, MDB, sham MDB	Self-administered 15 min, twice daily for 2 weeks; 25 Hz for optimal stimulation, pulse width 250 μs, intensity 0.1 to 10 mA (based on uncomfortable tingling sensation). Stimulation alternates between active cycles for 30 s, followed by a break of 30 s. Location: concha of the left ear.	Sham taVNS (The bipolar stimulation electrode is turned 180° and placed over the center of the left earlobe instead of the outer auditory canal; and sham MDB (focused paced breathing (22, 34, 35) at 12 breadths/min)	No significant change in HRV (primary outcome); trends toward reduced FM severity in active groups
Dolcini et al., 2025 [30]	Italy	Single-arm open-label pilot	FM	18	42.1 ± 11.6	70%	Auricular Vagal Neuromodulation Therapy (AVNT™)	30 min sessions, 5×/week for 4 weeks. Frequency within 1–30 Hz. Pulse width within 50–250 μs. Intensity could be adjusted from 0.1 to 36 mA based on the pain threshold. Location: tragus of the left ear.	No control group	Significant improvements in rFIQ and PSQI (symptoms and sleep); no significant change in BDNF levels

Abbreviations: AVNT™, Auricular Vagal Neuromodulation Therapy; BDNF, brain-derived neurotrophic factor; FM, fibromyalgia; HRV, heart rate variability; MDB, meditative-based diaphragmatic breathing; PSQI, Pittsburgh Sleep Quality Index; rFIQ, Revised Fibromyalgia Impact Questionnaire; RCT, randomized controlled trial; taVNS, transcutaneous auricular vagus nerve stimulation;VNS, vagus nerve stimulation.

**Table 2:** Study-level FMS outcomes assessment across included trials.

Study, Year	Measure	Results Mean Diff. ± SD	Key Methodological Limitations
Lange et al., 2011 [29]	NRS	Reduction in pain intensity observed; 36% (5/14) achieved ≥30% pain reduction at 3 months and 50% (7/14) at 11 months	Small sample; open-label design; no control group
Kutlu et al., 2020 [31]	VAS	Within-group significant improvements in pain. No difference between groups after treatment (Exercise: 3.45 ± 1.73; taVNS + exercise: 2.56 ± 1.91)	Between-group differences not statistically significant; short duration
Paccione et al., 2022 [32]	NRS	Within-group improvements in pain. No difference between groups (Δ pain: active tVNS = −0.57; sham tVNS = −0.86; active MDB = −0.59; sham MDB = −0.33)	Short duration; HRV measure may lack sensitivity
Dolcini et al., 2025 [30]	VAS	Significant improvement in FMS symptoms (FIQ baseline = 72.1 ± 12.8, FIQ 4-week follow-up = 55.1 ± 18.1) and Sleep quality (PSQI baseline = 13.1 ± 3.11, PSQI 4-week follow-up = 9.22 ± 3.78).	No control group; small sample; mechanistic data limited

Abbreviations: NRS, Numeric Rating Scale; VAS, Visual Analog Scale; taVNS, transcutaneous auricular vagus nerve stimulation; tVNS, transcutaneous vagus nerve stimulation; MDB, meditative diaphragmatic breathing; HRV, heart rate variability; FMS, fibromyalgia; PSQI, Pittsburgh Sleep Quality Index; Δ, change from baseline; SD, standard deviation.

**Table 3:** Published Study Protocols.

Study, Year	Country	Study Design	Study Population	Sample Size	Intervention Type	Control	Stimulation Protocol	Outcome Measures
Molero-Chamizo et al., 2022 [34]	Spain	Double-blinded RCT	FMS patients, aged 18–69	136	Non-invasive cervical & auricular VNS	Sham cervical and auricular VNS. Axillary nerve stimulation.	5 sessions/week × 4 weeks	Pain, fatigue, sleep, depression, HRV, and cytokines; compare stimulation sites
Gebre et al., 2018 [35]	United States	Randomized, single-blinded feasibility trial	Veterans with FMS, aged 20–60	20	Auricular percutaneous electrical neural field stimulation (PENFS) via the Neuro-Stim System (NSS) targeting auricular branches of cranial nerves including the vagus nerve.	Standard care comparator	NSS device worn 5 days, then weekly for 4 weeks; Control = individualized standard therapy (anticonvulsants, NSAIDs, topical agents, PT).	Feasibility of fcMRI as a biomarker of pain-related neural substrates during PENFS and whether PENFS improves pain and function vs. standard therapy. Primary outcome: DMN–insula resting connectivity; secondary: DVPRS pain, PROMIS/function tests, and analgesic use.

Abbreviations: FMS, fibromyalgia syndrome; RCT, randomized controlled trial; PENFS, percutaneous electrical neural field stimulation; NSS, Neuro-Stim System; HRV, heart rate variability; fcMRI, functional connectivity magnetic resonance imaging; DMN, default mode network; DVPRS, Defense and Veterans Pain Rating Scale; PROMIS, Patient-Reported Outcomes Measurement Information System; NSAIDs, non-steroidal anti-inflammatory drugs; PT, physical therapy.

## 4. Discussion

### 4.1. The Advantages of taVNS

taVNS provides a distinctly divergent approach when compared with conventional pharmacotherapy. While medications can be effective in treating disorders such as depression, anxiety, epilepsy, and inflammatory conditions, they frequently result in adverse effects, issues of tolerance, and variable long-term adherence [4]. Invasive cervical VNS (cVNS) is associated with a range of potential risks and complications, including surgical risks, infections, and voice changes [4,36].

In contrast, taVNS is non-invasive and delivered through electrodes on the external ear that activate the auricular branch of the vagus nerve. Its safety profile is well-established, and its administration is generally straightforward. Adverse effects are reported in a low percentage of cases [20]. Its home-based application has been demonstrated to promote enhanced patient adherence and autonomy when compared with clinic-based interventions [9].

taVNS has been demonstrated to enhance autonomic regulation, as evidenced by an augmentation in heart rate variability (HRV), and to engender quantifiable anti-inflammatory effects, manifesting as diminished TNF-α and IL-6 levels [37]. Recent studies employing functional imaging techniques have indicated that taVNS modulates neural circuits implicated in mood and cognitive function, including the nucleus tractus solitarius and limbic structures. Moreover, the taVNS application in health subjects has shown an increase in pain inhibitory tonus indexed by conditioned pain modulation [38], suggesting a direct mechanism influence in pain homeostatic processes. These findings also support the integration of taVNS with behavioral therapies, such as cognitive behavioral approaches, which underscore the potential benefits of combining these complementary therapeutic modalities with taVNS [27]. A detailed description of the taVNS procedure combined with multimodal biomarkers is presented in our previous publication [39].

Importantly, the most clinically realistic near-term role for nVNS in FM is as an adjunct within multimodal care (e.g., alongside exercise, sleep interventions, and psychological therapies), consistent with contemporary guideline emphasis on combined non-pharmacologic strategies; early FM trials suggest that taVNS can be feasibly combined with behavioral/rehabilitation approaches and may enhance improvements in function and symptom severity, even when between-group differences are modest [31,32]. Regarding contraindications and precautions, evidence from the broader neuromodulation and taVNS literature supports careful screening and clinician oversight in individuals with implanted electrical devices (e.g., pacemakers/ICDs), significant cardiac arrhythmias, or other conditions where autonomic stimulation could pose risk; additionally, pregnancy remains a common precautionary exclusion in device labeling and trials due to limited safety data [40,41].

Looking forward, the technology is evolving toward wearable and closed-loop systems. Prototype devices now integrate real-time physiological monitoring (e.g., HRV, skin conductance) to dynamically adjust stimulation parameters based on individual response [39,42]. Standardization efforts are also underway to optimize personalized stimulation protocols, encompassing frequency (commonly 20–25 Hz), pulse width (~0.25–1 ms), intensity (based on individual tolerance), timing (e.g., diurnal patterns), and ear side (left vs. right), tailored to specific clinical conditions [43].

### 4.2. Challenges and Future Directions

Despite promising early evidence, the field of VNS for FMS remains in a nascent phase, marked by key gaps in trial design, mechanistic understanding, and clinical translation. Addressing these limitations is critical to advance the development of VNS as a credible therapeutic modality for chronic nociplastic pain [40].

First, there is a pressing need for large-scale, sham-controlled randomized controlled trials (RCTs) with long-term follow-up. Most existing studies are limited by small sample sizes, short intervention durations (2–4 weeks), and inconsistent outcome measures. For example, while Paccione et al. (2022) implemented a rigorous four-arm RCT design, the trial lasted only 14 days and utilized ultra-short-term heart rate variability (HRV) as the primary outcome, which may be insufficient to detect sustained neurophysiological changes [32]. Similarly, Kutlu et al. (2020) and Dolcini et al. (2025) provided useful pilot data but were constrained by modest statistical power and open-label designs [30,31]. Moving forward, studies such as the Molero-Chamizo et al. (2022) protocol—testing both cervical and auricular tVNS in 136 patients with multi-dimensional outcomes—represent a more robust model for clinical evaluation [34].

Second, there is a lack of standardization in stimulation parameters, including site (auricular vs. cervical), frequency (Hz), pulse width, session duration, treatment length, and waveform characteristics [44]. Without harmonization across trials, it becomes difficult to compare outcomes or draw generalizable conclusions. The therapeutic window for VNS in FM is likely narrower and more individualized than in epilepsy or depression, requiring careful titration. The field would benefit from consensus guidelines on stimulation protocols derived from dose-finding or adaptive design studies.

Third, the identification of responders versus non-responders remains an unresolved issue. FM is a heterogeneous condition, with overlapping clusters of symptoms across somatic, cognitive, and affective domains [4]. Biomarkers such as baseline HRV, psychological profile, or inflammatory markers (e.g., IL-6, TNF-α, CRP) may help predict who benefits most from VNS. Dolcini et al. (2025) explored BDNF levels but found no significant modulation—suggesting that peripheral biomarkers alone may be insufficient [30]. More sophisticated responder profiling, possibly including network modelling of health outcomes and machine learning approaches to predict treatment response based on baseline features, may improve clinical precision.

Fourth, mechanistic studies using advanced neuroimaging or electrophysiological tools are lacking. Functional MRI (fMRI) studies could help map how taVNS affects connectivity in pain-related networks (e.g., anterior cingulate cortex, insula, amygdala), while EEG could track frequency-domain changes related to vagal tone and cortical excitability [45]. Cytokine profiling and gene expression analyses could further elucidate downstream anti-inflammatory or neuroplastic effects. Importantly, such mechanistic investigations may help distinguish specific neuromodulatory actions of VNS from non-specific placebo responses—a common concern in non-invasive device trials [46,47].

To advance the field, interdisciplinary collaboration among clinical pain researchers, neuroimaging experts, bioengineers, and computational modelers is essential. A comprehensive, multimodal research strategy is the only path to clarify how, for whom, and under what conditions VNS offers true therapeutic benefit in FMS.

From a pragmatic perspective, an ideal next-generation clinical trial to clarify the efficacy and mechanism of VNS in FMS would consist of a multi-center, double-blind, sham-controlled randomized trial with sufficient power and longitudinal follow-up. Such a trial should enroll a well-characterized FM cohort (n ≥ 150–200) and compare standardized auricular taVNS versus sham stimulation over a minimum active treatment period of 8–12 weeks, followed by a maintenance or observational phase extending to 6–12 months, to assess the durability of effects. Stimulation parameters should be pre-specified and harmonized (e.g., left cymba conchae stimulation, 20–25 Hz, pulse width 250–500 μs, intensity titrated to sensory threshold, 15–30 min once or twice daily), in accordance with existing consensus recommendations [44]. Importantly, the sham taVNS condition should be carefully designed to maintain blinding while minimizing vagal engagement, for example, by positioning identical placement to a non-vagally innervated auricular site (e.g., earlobe or outer helix), using inactive electrodes that reproduce cutaneous sensations without activating vagal afferents. Such approaches have been shown to improve blinding credibility while reducing the risk of physiological contamination of the control condition [46,47].

Primary outcomes should include patient-centered clinical endpoints such as pain intensity, functional impact (FIQR), fatigue, and sleep quality, while secondary outcomes should incorporate mechanistic biomarkers, including heart rate variability, inflammatory cytokines (e.g., IL-6, TNF-α), and neurophysiological or neuroimaging markers of network modulation [45]. Baseline phenotyping (autonomic tone, psychological profile, symptom clusters) should be integrated to enable responder stratification and predictive modeling, potentially supported by machine-learning approaches. Together, such a design would move the field beyond proof-of-concept toward definitive evidence regarding efficacy, mechanisms, optimal dosing, and patient selection for VNS in FMS.

### 4.3. Potential New Approach in FMS Management

FMS remains one of the most clinically challenging chronic pain conditions, largely due to its multifaceted pathophysiology and poor response to conventional pharmacological treatments. As such, there is an urgent need for mechanism-based, non-pharmacologic interventions that can address the dysregulated systems underlying nociplastic pain. In this context, taVNS exemplifies a potential innovation—one that aligns with a bioelectronic medicine paradigm capable of reshaping how we conceptualize and manage FM [48].

Bioelectronic medicine refers to the convergence of neuroscience, bioengineering, and digital health to develop targeted neuromodulation therapies that engage specific neural circuits for disease modification [49]. TaVNS fits squarely within this framework. By activating afferent fibers that project to the NTS and then to higher-order brain regions involved in pain, emotion, and autonomic regulation, taVNS holds the potential to recalibrate central sensitization, modulate inflammatory responses, and restore autonomic balance—three hallmarks of FMS pathophysiology [50,51].

Crucially, taVNS is non-invasive, scalable, and well-tolerated, making it highly suitable for integration into home-based, multimodal care models. Emerging data suggest that taVNS can complement rehabilitation programs, cognitive behavioral therapy, and exercise interventions—offering synergistic effects that may enhance adherence and outcomes. Furthermore, taVNS is compatible with digital therapeutics, enabling closed-loop or algorithm-guided delivery based on real-time physiological feedback (e.g., HRV, sleep metrics). Future iterations could incorporate wearable sensors and AI-driven personalization to optimize stimulation parameters and improve engagement.

The integration of taVNS into FM care also encourages a change in how both clinicians and patients conceptualize treatment: from symptom suppression to network physiology modulation, from reactive pharmacology to proactive self-regulation, and from fragmented management to biopsychophysiological integration. In this sense, VNS does not merely offer a new tool—it could offer a new management mindset.

If validated by ongoing trials and mechanistic studies, taVNS and other non-invasive vagal stimulation methods may serve as cornerstones in a new class of complexity-oriented digital bioelectronic interventions for FM and other central sensitivity syndromes. Such a shift could bring pain medicine closer to precision, scalability, and sustainability—goals long overdue in the management of chronic pain.

## 5. Conclusions

FMS represents a disorder of disrupted network physiology spanning autonomic, immune, and central nervous system domains, for which current symptom-focused therapies provide limited benefit. The available evidence suggests that vagus nerve stimulation—particularly non-invasive transcutaneous auricular VNS—may favorably modulate these interconnected systems and improve key clinical outcomes. However, the existing literature is constrained by methodological heterogeneity and limited trial duration. Future rigorously designed, sham-controlled studies incorporating standardized stimulation protocols, mechanistic biomarkers, and responder stratification are essential to determine the clinical utility of VNS. If validated, network-informed vagal neuromodulation could represent a scalable, mechanism-based therapeutic strategy for FMS and related central sensitization syndromes.

## Data Availability

No new data were generated for this review.

## Supplementary Materials

The following supporting information can be downloaded at: https://www.mdpi.com/article/10.3390/jfmk11010015/s1, Table S1: Search strategy.
