# Supplementary material for: Potential of Vagus Nerve Stimulation to Modulate Fibromyalgia’s Network Physiology: A Systematic Review
Source: J Funct Morphol Kinesiol. 2025 Dec 29;11(1):15. doi: 10.3390/jfmk11010015 (PMC12821727; doi:10.3390/jfmk11010015)
Supplement: Supplementary file 1 [file jfmk-11-00015-s001.zip › jfmk-4030794-supplementary.pdf]

Supplementary Materials

Table S1. Search strategy.

Pubmed

(("Fibromyalgia"[Mesh] OR fibromyalgia[tiab] OR "fibromyalgia syndrome"[tiab] OR FMS[tiab])) AND (("Vagus Nerve Stimulation"[Mesh] OR "vagus nerve stimulation"[tiab] OR VNS[tiab] OR "vagal nerve stimulation"[tiab] OR "transcutaneous vagus nerve stimulation"[tiab] OR "transcutaneous auricular vagus nerve stimulation"[tiab] OR taVNS[tiab] OR tVNS[tiab] OR "auricular vagus nerve stimulation"[tiab] OR "auricular vagal stimulation"[tiab] OR "cervical vagus nerve stimulation"[tiab] OR "non-invasive vagus nerve stimulation"[tiab] OR nVNS[tiab] OR "implanted vagus nerve stimulation"[tiab] OR "vagus nerve"[TIAB] OR "vagal tone"[TIAB] OR "parasympathetic"[TIAB]))

Embase

((Fibromyalgia/exp OR fibromyalgia:ti,ab OR 'fibromyalgia syndrome':ti,ab OR FMS:ti,ab)) AND (('Vagus Nerve Stimulation'/exp OR 'vagus nerve stimulation':ti,ab OR VNS:ti,ab OR 'vagal nerve stimulation':ti,ab OR 'transcutaneous vagus nerve stimulation':ti,ab OR 'transcutaneous auricular vagus nerve stimulation':ti,ab OR taVNS:ti,ab OR tVNS:ti,ab OR 'auricular vagus nerve stimulation':ti,ab OR 'auricular vagal stimulation':ti,ab OR 'cervical vagus nerve stimulation':ti,ab OR 'non-invasive vagus nerve stimulation':ti,ab OR nVNS:ti,ab OR 'implanted vagus nerve stimulation':ti,ab OR 'vagus nerve':ti,ab OR 'vagal tone':ti,ab OR parasympathetic:ti,ab))

Cochrane Central

(([mh Fibromyalgia] OR fibromyalgia:ti,ab OR "fibromyalgia syndrome":ti,ab OR FMS:ti,ab)) AND (([mh "Vagus Nerve Stimulation"] OR "vagus nerve stimulation":ti,ab OR VNS:ti,ab OR "vagal nerve stimulation":ti,ab OR "transcutaneous vagus nerve stimulation":ti,ab OR "transcutaneous auricular vagus nerve stimulation":ti,ab OR taVNS:ti,ab OR tVNS:ti,ab OR "auricular vagus nerve stimulation":ti,ab OR "auricular vagal stimulation":ti,ab OR "cervical vagus nerve stimulation":ti,ab OR "non-invasive vagus nerve stimulation":ti,ab OR nVNS:ti,ab OR "implanted vagus nerve stimulation":ti,ab OR "vagus nerve":ti,ab OR "vagal tone":ti,ab OR parasympathetic:ti,ab))
